# Serious Concern of Congenital Zika Syndrome (CZS) in India: A Narrative Review

**DOI:** 10.1155/2024/1758662

**Published:** 2024-06-13

**Authors:** Maneesh Kumar, Suman Kumar, Ratnesh Kumar, Mithilesh Kumar Jha, Shashank Nand Tiwari, Pratima Gupta

**Affiliations:** ^1^ State-Viral Research and Diagnositic Laboratory Department of Microbiology All India Institute of Medical Sciences, Deoghar 814152, Jharkhand, India; ^2^ Department of Microbiology All India Institute of Medical Sciences, Deoghar 814152, Jharkhand, India

**Keywords:** congenital abnormalities, congenital Zika syndrome, microcephaly, pregnant women, Zika virus

## Abstract

Congenital Zika syndrome (CZS) is a major concern in India and highlights the multifaceted challenges posed by the Zika virus (ZIKV). The alarming increase in CZS cases in India, a condition that has serious effects on both public health and newborns, has raised concerns. This review highlights the importance of raising concern and awareness and taking preventive measures by studying the epidemiology, clinical symptoms, and potential long-term consequences of CZS. The review also contributes to worldwide research and information sharing to improve the understanding and prevention of CZS. As India deals with the changing nature of CZS, this thorough review is an important tool for policymakers, health workers, and researchers to understand what is happening now, plan for what to do in the future, and work together as a team, using medical knowledge, community involvement, and study projects to protect newborns' health and reduce the public health impact of these syndromes.

## 1. Introduction

The Zika virus (ZIKV) is one of the latest invaders to cause global concern. Its rapid spread, increasing virulence, and complex clinical symptoms have caused much fear and alarm. It causes a mild fever and nausea, similar to dengue fever. Microcephaly in newborns and Guillain–Barré syndrome (GBS) are two neurological manifestations often associated with severe courses of this disease [1, 2]. The causative virus was first discovered in 1947 as part of the yellow fever transmission surveillance program in Uganda. Later, the virus was detected in several African and Southeast Asian countries, causing mild cases. Its severe outbreak in Brazil in 2015–2016 had attracted worldwide attention [3]. Complicated clinical outcomes occurred in many countries as ZIKV became more contagious and spread rapidly, making people worried and anxious [4, 5]. The problem is worsening worldwide because people do not have enough advanced information and effective prevention and treatment methods.

Like most flaviviruses, ZIKV is mainly spread by *Aedes* mosquitoes [6]. The transmission cycles of ZIKV are characterised by sylvatic transmission, that is, between the vector, a hematophagous mosquito, and the primary host, a vertebrate. Urban transmission has also been observed to play an important role in the spread of the virus and often leads to epidemics [7]. In addition, there are a number of other known transmission routes for ZIKV that have only recently come to light which include intrauterine transmission, sexual intercourse, and transmission through blood. Individuals must follow proper safety measures and be very careful with blood transfusions, organ donations, and sexual activity [6, 8, 9].

Given the propensity of ZIKV to cause asymptomatic infections, we cannot ignore the history of transmission. Changes in ZIKV over time may have led to the cases in Rajasthan and Madhya Pradesh, mirroring those observed in Yap Island and French Polynesia. *Aedes aegypti* is prevalent in certain areas of Jaipur City, such as Shastri Nagar and Rajput Hostel, suggesting that it could be involved in the transmission of ZIKV during the current outbreak. Researchers have also linked *Ae. albopictus*, *Ae. hensilli* on the island of Yap, and *Ae. polynesiensis* in French Polynesia to the spread of ZIKV. ZIKV can be transmitted from person to person in many ways, including sexual contact, vertical transmission, peripartum transmission, blood transfusions, monkey bites, and laboratory diseases [10]. Previous ZIKV infections in Gujarat (2016) and Tamil Nadu (2017) and the 2018 outbreak in Jaipur affected people who had never been outside India or to other states [11, 12]. This indicates that the Asian strain is spreading within India. The 1954 report of the Indian Council of Medical Research–National Institute of Virology (ICMR-NIV) in Pune, which found ZIKV seropositivity in 16.8% of samples from various Indian locations, supports this view. Researchers have detected the ZIKV in Gujarat, Tamil Nadu, and Rajasthan, suggesting that it has been present in the country for a long time. Cases of ZIKV have even been reported in Bangladesh, a neighbouring country [13].

For the first time in the history of the virus, pregnant women in Brazil were infected with ZIKV. Initial reports stated that 35 babies born between August and October 2015 were diagnosed with microcephaly, as the virus transmission caused a series of congenital abnormalities in babies born to mothers who had Zika fever during pregnancy. It was found that the mothers either lived in ZIKV-affected areas during this time or travelled there during pregnancy. Twenty-five (71%) of these 35 newborns had severe microcephaly, 17 (49%) had at least one neurological abnormality, and all babies who underwent clinical examination had behavioural abnormalities [14, 15]. Later, in 2016, such abnormalities were defined as congenital Zika-associated syndrome by world-leading institutes, that is, the Centres for Disease Control and Prevention (CDC) and the World Health Organization (WHO) [16]. From then until 2020, the Brazilian Ministry of Health received 3523 confirmed cases of congenital Zika syndrome (CZS). This review focuses on the prevalence of ZIKV infection in pregnant women in India and the potential risks to newborns and breastfeeding mothers, as well as the future occurrence of CZS.

## 2. CZS Detection in Pregnant Women and Government Concern

Certain epidemiologic data on the management of Zika infections in India may have been difficult to obtain at the time of the last update in January 2022 [17]. On the other hand, India's Zika infection control generally includes surveillance, vector control, public awareness program, clinical management, and collaboration with other countries. To better understand the spread of the virus, surveillance includes case detection, cluster identification, and trend monitoring.

Retrospective surveillance for this study shows that the ZIKV has spread to previously unaffected areas. This indicates local transmission of the virus within India. Reports of the presence of ZIKV in 16 Indian states and Union Territories (UTs) between 2017 and 2021 indicate an increasing prevalence of the virus [18]. Specialists from the ICMR have confirmed occurrences of ZIKV in Telangana, Jharkhand, Rajasthan, Punjab, Delhi, and other UTs in 2021. In 2017, incidents were reported in Gujarat and Tamil Nadu, and in 2018, they were also reported in Rajasthan and Madhya Pradesh. The regional distribution of ZIKV indicates that the virus is not spreading evenly across the country [12]. The presence of the virus in these areas indicates ecological and environmental conditions that favour the proliferation of *Aedes* mosquitoes, the main vectors of ZIKV [19]. In addition, travel habits, population density, and urbanisation could play a role in the spread of the virus in these locations. Effective surveillance and control methods are crucial to prevent further spread of the virus and mitigate its harmful impact on public health. Health authorities must be vigilant to stop the spread of ZIKV in affected areas and take targeted measures [6].

**Figure 1:**
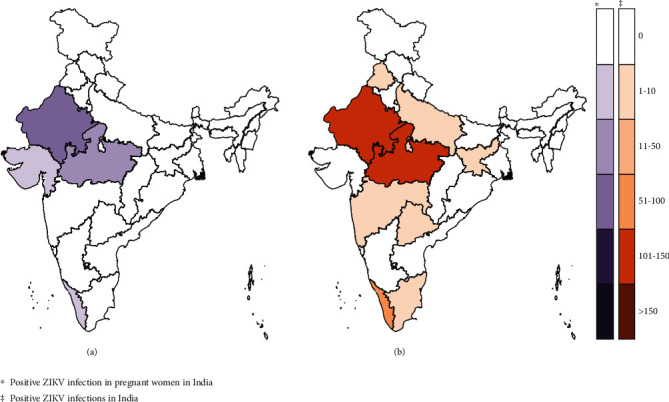
Different regions of India got infected by Zika in pregnant women and a Zika infection to all individuals. The epidemiologic prevalence of ZIKV infection in pregnant women and overall positive cases in all regions of India illustrate the rate and distribution of the virus in this vulnerable group and the general population. It provides important insights into regional infection patterns that help in targeted public health interventions and resource allocation.

Insecticides and the reduction of breeding sites are the most important vector control tools to prevent the spread of ZIKV by the *Aedes* mosquito [20]. Public education campaigns inform people about the ZIKV, its symptoms, and how they can protect themselves by applying insect repellent and wearing protective clothing. Pregnant women are given special attention due to the increased risk of birth defects associated with ZIKV infection. Clinical management focuses on the detection and treatment of Zika cases. Patients are often given supportive treatment to alleviate symptoms. As a bonus, India is working with global groups such as the WHO to share knowledge and resources for better treatment methods [21]. Current and comprehensive epidemiological data on the management of Zika infections in India can best be found in the official reports and publications of the Ministry of Health and Family Welfare or other relevant health authorities [11, 20]. The National Centre for Disease Control (NCDC) is responsible for managing the ZIKV in India as an outbreak-tracking hub, with the NIV and the NCDC serving as reference laboratories for diagnosis [22, 23]. The ICMR has recognized 10 national virology laboratories for Zika testing. The Ministry of Health has set up a joint surveillance group to monitor the Zika situation in India and advise pregnant women to avoid travelling to affected areas (Figure 1). Various preventive measures, such as vaccine development, implementation of vector control program, and public awareness, are being taken to combat viral infection in India [20, 24–26].

## 3. Serious Concern About CZS

In light of the increasing concern about CZS, the WHO declared ZIKV infection a Public Health Emergency of International Concern on 28 January 2016 and recommended that all countries establish surveillance systems for ZIKV and CZS [21, 27]. CZS includes congenital malformations such as arthrogryposis, brain calcifications, exaggerated primitive reflexes, hydrocephalus, joint deformities, hyperexcitability, and irritability. In some cases, the patient's head is disproportionately smaller than that of most people of his or her age and sex, and the brain grows inadequately. Unfortunately, around 90% of infected individuals have difficulties learning and thinking for this reason [28, 29]. In severe cases, mental retardation, movement disorders, balance disorders, convulsions, hearing and vision disorders, and joint inflammation can occur. Among the many medical problems associated with CZS, these symptoms fall into a subgroup. It is important to note that microcephaly is a clinical observation and not a disease. It is an abnormally small head size that is often associated with neurological effects and developmental problems in affected individuals. Such problems can lead to miscarriages that later require additional research and interventions to understand and control the virus [30–32]. Eliminating unfavourable conditions and introducing improved prevention, management, and treatment methods for microcephaly would improve precision. Between 2013 and 2014, an outbreak of the ZIKV swept through the tropical regions and mainly affected numerous islands in the southern Pacific, where a high mortality rate was recorded. Later, in 2015, indigenous transmission was documented in Brazil. The confirmation of local transmission in Brazil was a pivotal moment in understanding the ability of the virus to establish itself in new regions and prompted increased surveillance, research, and public health efforts to combat the spread of the virus and mitigate its impact [33]. The virus has caused a host of economic and social problems that have had a profound impact on the families and mothers of children diagnosed with ZIKV, as well as on healthcare organizations [16].

The clinical manifestation of the ZIKV is responsible for many public health implications. Although pregnant women often show no symptoms of ZIKV, the incubation period until the onset of infection can be 3 to 12 days. In India, public awareness of Zika is low compared to other viral infections [34, 35]. For this reason, ZIKV could spread without proper surveillance and diagnosis among the infected people. The biggest concern for expectant mothers is the likelihood of Zika infection, which can lead to glaucoma, optic neuropathy, lissencephaly, and ventriculomegaly [36, 37]. Apart from this, in the study conducted by Carvalho et al., a 5%–13% chance of developing cerebral palsy in newborns was observed, with a risk of severe brain abnormalities and collateral retinal scarring in 3%–9% of cases [38]. Furthermore, they found no signs of microcephaly on sonographic examination in any of the newborns of recently delivered women or pregnant women. In contrast, Melo et al. documented 4 miscarriages out of 62 cases (6.4%) and 3 intrauterine deaths out of 62 cases (4.8%) during ZIKV infection [39]. No cases of microcephaly, morbidity, or GBS as a result of the viral infection were found in the expectant mothers themselves. However, it is important to note that these diseases are usually diagnosed in the developing fetuses and not in the mothers. This underscores the importance of prenatal screening and diagnostic measures for early detection of potential complications, allowing appropriate medical intervention and support for both the affected fetuses and the expectant mothers. Researchers from North and South America, the Pacific, and French Polynesia were confronted with the contrasting situation of ZIKV infection. They found that the virus was also associated with GBS, meningoencephalitis, and acute myelitis. Among the patients, 21% of the infants had eye abnormalities, 9% had a low gestational age, and 2% were stillborn. The risk of Zika-related damage to the fetus is highest in the first trimester of pregnancy, although it can also occur in the second or even early third trimester [10, 40, 41].

## 4. Prevalence and Surveillance of ZIKV in India

Indian health authorities have been monitoring the Zika situation since 2013, shortly after the first ZIKV outbreak occurred in the Marquesas Islands and later moved to Brazil in May 2015 [42, 43]. Circumstances changed when the WHO declared ZIKV a global health emergency (Table 1). The WHO classifies ZIKV infection into different categories depending on the severity of the symptoms and the possible complications associated with the virus.

At that time, the WHO classified India as Category 4, as there was a suspected case of the virus but no documented cases. With three confirmed cases, India is now classified as a WHO Category 2. Subsequently, Mourya et al. were exclusively involved in planning to review the epidemiologic situation and management options for ZIKV epidemics in India [22]. The national network of Viral Research and Diagnostic Laboratories (VRDLs) of the ICMR started ZIKV sentinel surveillance in March 2016 [45]. At an early stage of surveillance, four positive cases of ZIKV were confirmed in Chennai, India [11]. Against this backdrop, the NIV in Pune has scaled up its ZIKV testing laboratories and provided ZIKV molecular testing reagents to all VRDLs. From an initial 10 labs in 2016, the number of VRDLs increased to 56 in 2018 and 132 in 2021 for ZIKV surveillance. The VRDLs were instructed to test at least ten dengue and chikungunya virus (CHIKV)-negative samples for ZIKV each year. Although the COVID-19 pandemic is affecting the flow of ZIKV testing, the NIV requires only DENV/CHIKV negative samples from all VRDLs for self-testing via reverse transcription polymerase chain reaction (RT-PCR). Laboratory-based surveillance in 55 Indian tertiary facilities included screening for neonatal birth defects [21]. Researchers are then beginning to look at the incidence of ZIKV infection in newborns. The sudden outbreak of ZIKV in Gujarat (2016–2017), Tamil Nadu (2017), and Rajasthan and Madhya Pradesh (2018) is enough to alert Indian researchers to take decisive steps against the rare cases in newborns and pregnant women [10, 12, 46]. It is also extremely important not to lose sight of efficient methods of vector control and to focus on researching and developing a vaccine against ZIKV that could be effectively administered to pregnant women [18]. In addition to laboratory efforts, concerns have been raised about the widespread distribution of the causative vector for virus transmission and the susceptible host population [11, 47].

## 5. CZS in Newborns of Women Infected by ZIKV During Pregnancy

India confirmed its first ZIKV case in November 2016 in a woman after giving birth before she was discharged from the hospital. The case was detected during routine surveillance and tested for ZIKV. She has no symptoms such as a skin rash, joint pain, or digestive problems. Her blood sample was collected on November 14 and tested at the NIV in Pune, India. As part of the surveillance protocol, the woman was also tested for dengue, chikungunya, and ZIKV infections using standard RT-PCR techniques at the B.J. Medical College in Ahmedabad in 2016. This was the first confirmed case of Zika in India. Another 22-year-old pregnant woman was also found to be ZIKV positive at 37 weeks gestation, but the child was in good clinical condition at birth and no further follow-up was required [12]. The researchers emphasize the importance of postnatal follow-up. Despite the newborn showing a good clinical condition at birth, it is crucial to maintain monitoring of infants born to ZIKV-infected mothers into the postnatal period. This prolonged observation is essential due to the potential for delayed syndrome manifestations that might not be evident immediately after birth [48].

In response to this development, Gujarat undertook a concerted effort to screen pregnant women attending antenatal care (ANC) and those with high fever to identify possible cases in the state. This made it easier to find the cases reported in this investigation. Partial E-gene sequencing of the ZIKV genome was performed on a sample from the first case. This ZIKV type had more similarity to the virus found in *Aedes* mosquitoes in Malaysia in 1966 [12] and belonged to the Asian lineage than the variants that caused outbreaks between 2013 and 2016. All indications are that the ZIKV strain in India is indigenous to that country and was not introduced from elsewhere in South or Central America. It was also found that a specific strain of Asian ZIKV may already be circulating in India. Subsequently, there were sporadic cases of ZIKV in Krishnagiri (Tamil Nadu) in June 2017 and sporadic cases in Ahmedabad (Gujarat) in September to October 2018 [21].

ZIKV later broke out in two Indian states: Madhya Pradesh (from October 2018 to November 2018) and Rajasthan (from September to October 2018). In 2018, there were 159 confirmed cases of ZIKV in Rajasthan and 127 in Madhya Pradesh, affecting 63 and 42 pregnant women, respectively. The cases reported from Jaipur, Rajasthan, were contained through targeted vector control and case detection measures. This technique proved to be highly effective [49]. Ten different ZIKV strains were sequenced in whole or in part from samples collected at different stages of the epidemic in Rajasthan. The ZIKV strains found in Rajasthan (2018) and Gujarat (2017) differ from each other but share a common Asian origin. The strains from Rajasthan, on the other hand, occur only sporadically but are more closely related to the ZIKV strains that have caused outbreaks in Brazil, the USA, Guatemala, and other countries. According to the research, there are two different strains of ZIKV circulating in India. It is possible that this discovery indicates the introduction of a new, more dangerous strain of ZIKV from Asia with increased outbreak potential following the spread of a less dangerous indigenous strain in Gujarat in 1954. The second strain was most likely responsible for the larger epidemic [13, 21]. Between July 8 and July 26, 2021, 590 blood samples were collected in Kerala through active case finding and passive surveillance. At NIV Pune, RT-PCR confirmed the presence of ZIKV in 70 cases (11.9%), including five pregnant women. Except for two cases reported from Ernakulam and Kottayam districts, all cases were from Trivandrum district. However, the residents of these two areas had recently travelled to the Trivandrum district.

Considering the lack of testing facilities, cases without symptoms, and coinfections with similar diseases, the actual number could be much higher (as shown in Table 2). It is to be feared that a pandemic triggered by ZIKV will put further strain on an already overburdened healthcare system. The clinical course of the virus has a broad spectrum of effects on public health. Zika infection is usually asymptomatic, with an incubation period of 3 to 12 days [53]. This favours the spread of ZIKV in the community and thus increases the burden. Some infected individuals remained seropositive until Day 11, while the majority remained seropositive until Day 3 [53, 54]. A significant proportion of these symptoms are also observed in the clinical presentation of other arbovirus-mediated diseases, including malaria, dengue, and chikungunya, in addition to COVID-19. Malaria, for example, is a protozoan infection that is widespread in India. ZIKV disease resolves on its own and eventually along with GBS [53, 55]. It is therefore important to rule out ZIKV and COVID-19 in every patient with GBS.

Although the high population of *Aedes* mosquitoes in India makes ZIKV a major threat, the large number of asymptomatic patients and the inadequate diagnostic infrastructure make a correct diagnosis of ZIKV difficult. To reduce the impact of ZIKV, diagnostic capacity must be strengthened and vector control methods must be improved. Vigilance is crucial to quickly detect and treat outbreaks. We should also pay special attention to pregnant women, as ZIKV infection during pregnancy can lead to CZS. In India, GBS, another consequence of ZIKV, has not occurred, but CZS has [56]. To gain a complete understanding of these syndromes and their significance, it is crucial to closely monitor the clinical features and collect more data. India can be better prepared to address the problems caused by ZIKV and similar arboviral diseases by improving its diagnostic capabilities, employing effective vector control techniques, and closely monitoring vulnerable populations.

## 6. Government Initiatives to Combat CZS and Its Vector

The Indian government has recognized the serious threat to public health and has developed a comprehensive approach to combat CZS. Strong vector control programs are an important part of these steps as it is hoped to reduce the number of mosquitoes, especially the *Aedes* species that spread ZIKV. This requires a lot of work, such as spraying with larvicides, eliminating places where mosquitoes can breed, and developing programs that encourage people to use bed nets and insect repellents [8, 13]. This vector is prevalent in India due to poor water management, inadequate sanitation system, poor sanitation facilities, and lack of public knowledge about reproduction [57]. To stop the spread of ZIKV, it is important to adopt a broad-based approach that includes consistent vector investigation and combined vector control management. Surveillance is based on the biting and resting populations of adult mosquitoes and a range of larval indices [58–60]. For integrated vector management, changes are made to the environment; people are protected with protective clothing and insect sprays; *Bacillus thuringiensi*s and fish larvae are used for biological control; temephos, pyrethrum, and malathion are used for chemical control; and legislation is enacted.

CZS control in India largely depends on the specialised functions of the NCDC in Delhi and the NIV in Pune. The NCDC is the apex body responsible for disease surveillance, outbreak investigation, and coordination of public health measures [61, 62]. In this context, NCDC conducts active surveillance to monitor ZIKV transmission, communicates with state health departments to ensure rapid reporting of cases, and provides technical support for vector control measures. It plays an important role in capacity building by training medical personnel in the diagnosis and treatment of CZS [63]. The NIV, on the other hand, is an exceptional research institute that focuses on viral infections such as ZIKV. The NIV exclusively researches the epidemiology, pathogenesis, and diagnostics of ZIKV, which contributes to our understanding of the mechanism of the infection. The NIV is developing and validating a viral RT-PCR kit. This ZIKV detection kit is rigorously tested to ensure accuracy and reliability. After testing, the kit is sent to various schools in the ICMR network. These organizations use the kit to monitor the spread of the ZIKV in different parts of the world. The NIV uses this standard test to ensure uniform detection and diagnosis of ZIKV cases across the country. This project makes it easier for health facilities to quickly detect and respond to ZIKV outbreaks, which contributes to better management and control of the disease in the long term [59, 61, 64]. To keep up with the ever-changing infectious disease landscape, it is crucial to regularly update and change tactics based on new scientific information. With a thorough and ever-changing ZIKA prevention strategy, India can work towards reducing the impact of ZIKV on pregnant women and protecting future generations [63, 65]. Improving the fight against CZS aims to mitigate the negative impact on affected individuals and their communities. This means making it easier for healthcare organizations to stop, diagnose, and properly treat CZS cases. The goal is to reduce the impact of CZS complications on babies and their families by making it easier for women to access prenatal care, follow screening protocols, and receive comprehensive medical care. Community education and support program play a critical role in raising awareness of CZS, promoting precautions, and supporting affected families, thereby strengthening resilience and improving treatment outcomes.

## 7. Future Prospective of CZS

India has taken the initiative to track and control the spread of CZS, focusing particularly on pregnant women. The country has also taken measures to curb the spread of the *Aedes* mosquito and educate the public about the virus. It is important to remain vigilant to quickly detect and control potential outbreaks to reduce the risk of ZIKV. Prevention strategies, such as avoiding mosquito bites and safe sex practices, can be further strengthened for pregnant women through efforts to improve medical facilities, accessibility, and education. Future results will depend heavily on collaboration between medical professionals, researchers, and health authorities. This includes both high-level initiatives, such as legislative efforts, and low-level positions within the health system, such as the employment of social health aides (ASHA) [52], as they have the potential to play a crucial role in disseminating vital information to the most vulnerable populations. In the absence of viable treatment alternatives, it is essential that measures are taken to educate parents and health professionals about preventative measures and the characteristics of each condition.

There is currently no consensus on whether pregnant women or newborns should generally be screened for infections. Many factors play a role in screening for these diseases, including the mother's immune status, the baby's exposure, and the baby's clinical symptoms [63]. There is no recommendation not to breastfeed newborns, even if the virus is detected in breast milk. There is currently no approved vaccine against ZIKV. The impact of ZIKV disease on pregnant women and their babies has spurred the development of vaccines around the world. According to the WHO/UNICEF Target Product Profile of Zika Vaccines [65], the preferred ZIKV vaccine candidate should primarily be able to reduce the incidence of clinical ZIKV disease in persons aged 9 years and older and should be safe to administer during pregnancy. As suggested by Gupta et al., the vaccine should be administered subcutaneously or intramuscularly and have a shelf life of at least 12 months at −20°C or 6 months at 2–8°C. To ensure the safety of pregnant women, adjuvants that are nonreplicating or inactivated should be used. When preparing multiple-dose containers, the WHO protocol for multiple doses should be followed [21, 66, 67]. There is currently no conclusive treatment for CZS. To ensure timely treatment, it is important to recognize and closely monitor babies who show signs of developmental delays and sensory impairments as early as possible. Even if a cure for CZS is not yet possible, it is important to start supportive and symptomatic therapies as soon as possible. The aim of these therapies is to improve the quality of life of those affected and alleviate their symptoms [68, 69]. To improve outcomes for people affected by CZS, proactive monitoring and early intervention measures are crucial, emphasizing the importance of comprehensive treatment and continued research efforts.

## 8. Conclusion

ZIKV infects pregnant women and transmits them to their unborn children at any time, especially during the prenatal and perinatal periods. Congenital ZIKV infections can have serious consequences for babies. Finally, the fact that CZS has been found in India shows how important it is to keep an eye on public health. Vector control and public education are two of the most important measures to prevent the spread of ZIKV. We can improve our understanding of how outbreaks or epidemics originate, evolve, and impact communities around the world by fostering international research collaborations and putting best practices into practice. This information is critical to the development of future strategies to effectively contain and manage similar public health problems. A multifaceted approach that includes medical interventions, community involvement, and ongoing research is critical to reduce the long-term impact of this congenital disease. Pregnant women in particular should take care of their own cleanliness and hygiene when in ZIKV-infected regions. Applying repellents, using mosquito nets, and wearing protective clothing are some of the preventative measures that can be taken to significantly reduce the risk of transmission. Given the potential damage that ZIKV can cause to the developing fetus, pregnant women should exercise caution when taking these measures. People need to focus on the prevention methods that can protect their health and prevent the spread of the virus by prioritizing preventative measures. This underscores the need for aggressive public health programs in the fight against infectious diseases like Zika. The focus must be on increased ZIKV surveillance, which would also help in the prevention of viral infection. Healthcare workers should also be involved in educating and monitoring pregnant women to reduce the incidence of ZIKV. To keep up with the ever-changing infectious disease landscape, it is crucial to regularly update and change tactics based on new scientific information. With a thorough and ever-changing ZIKA prevention strategy, India can work towards reducing the impact of ZIKV on pregnant women and protecting future generations.

## Figures and Tables

**Table 1:** WHO classifies Zika virus infection [44].

**Classifications**	**Symptoms**	**Potential complications**
Category 1	Asymptomatic or mild symptoms	People who have contracted the Zika virus may have no symptoms at all or only mild symptoms such as fever, skin rash, joint pain, and conjunctivitis (red eyes). In most cases, these symptoms disappear on their own without causing any major problems.
Category 2	Symptomatic Zika virus disease	This category includes people who have not only mild but also more serious illnesses such as fever, skin rash, joint pain, and conjunctivitis but who do not require hospitalization. Although the signs can be painful, they do not usually get worse and do not cause serious health problems.
Category 3	Zika virus disease with complications	There is a group of people who have been infected with the Zika virus and subsequently developed complications. These problems can include Guillain–Barré syndrome (GBS), an autoimmune disease that can lead to muscle weakness or paralysis, or microcephaly and other neurological problems in newborns if the infection occurs during pregnancy.
Category 4	Severe Zika virus disease	This category includes people with severe symptoms that require hospitalization or intensive care. Some of these signs could be neurological problems such as encephalitis or meningitis, which can be very dangerous to your health and may require special treatment.

**Table 2:** Positive ZIKV infection in pregnant women in India.

**State**	**No. of pregnant women positive for ZIKV**	**Year**	**References**
Gujarat	1	2016	[12]
Gujarat	1	2017	
Rajasthan	63	2018	[20, 37, 50]
Madhya Pradesh	34	2018	
Kerala	5	2021	[51, 52]

## Data Availability

Data sharing is not applicable to this article as no datasets were generated or analysed during the current study.
